# Nanoparticles functionalized with stem cell secretome and CXCR4-overexpressing endothelial membrane for targeted osteoporosis therapy

**DOI:** 10.1186/s12951-021-01231-6

**Published:** 2022-01-15

**Authors:** Chi Zhang, Wei Zhang, Dashuai Zhu, Zhenhua Li, Zhenzhen Wang, Junlang Li, Xuan Mei, Wei Xu, Ke Cheng, Biao Zhong

**Affiliations:** 1 Department of Orthopedics, Shanghai Jiao Tong University Affiliated Sixth People’s Hospital, 600 Yishan Road, Shanghai, 200233 China; 2 Joint Department of Biomedical Engineering, The University of North Carolina at Chapel Hill and North Carolina State University, Chapel Hill, NC USA; 3 Department of Orthopedics, Tongren Hospital, Shanghai Jiao Tong University School of Medicine, 1111 XianXia Road, Shanghai, 200336 China

**Keywords:** Osteoporosis, CXCR4, Secretome, Mesenchymal stem cells, Bone targeting, Nanoparticles

## Abstract

**Background:**

Osteoporosis is a chronic condition affecting patients’ morbidity and mortality and represents a big socioeconomic burden. Because stem cells can proliferate and differentiate into bone-forming cells, stem cell therapy for osteoporosis has been widely studied. However, cells as a live drug face multiple challenges because of their instability during preservation and transportation. In addition, cell therapy has potential adverse effects such as embolism, tumorigenicity, and immunogenicity.

**Results:**

Herein, we sought to use cell-mimicking and targeted therapeutic nanoparticles to replace stem cells. We fabricated nanoparticles (NPs) using polylactic-co-glycolic acid (PLGA) loaded with the secretome (Sec) from mesenchymal stem cells (MSCs) to form MSC-Sec NPs. Furthermore, we cloaked the nanoparticles with the membranes from C–X–C chemokine receptor type 4 (CXCR4)-expressing human microvascular endothelial cells (HMECs) to generate MSC-Sec/CXCR4 NP. CXCR4 can target the nanoparticles to the bone microenvironment under osteoporosis based on the CXCR4/SDF-1 axis.

**Conclusions:**

In a rat model of osteoporosis, MSC-Sec/CXCR4 NP were found to accumulate in bone, and such treatment inhibited osteoclast differentiation while promoting osteogenic proliferation. In addition, our results showed that MSC-Sec/CXCR4 NPs reduce OVX-induced bone mass attenuation in OVX rats.

**Graphical Abstract:**

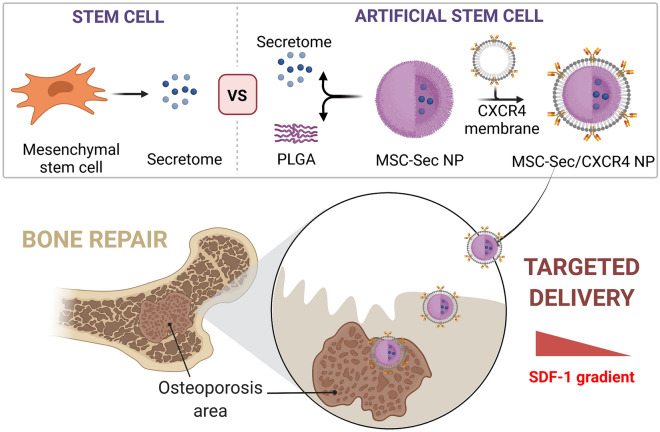

**Supplementary Information:**

The online version contains supplementary material available at 10.1186/s12951-021-01231-6.

## Background

Osteoporosis is recognized by decreased bone mass and degraded bone microstructure due to an imbalance of osteoclasts and osteoblasts [1–3]. This disease affects millions of people worldwide with increased bone fragility and risk of bone fracture. Bisphosphonates, a class of drugs that increase bone mineral density, have been widely used in the treatment of osteoporosis [4]. However, there are potential side effects such as increased risk of osteonecrosis of the jaw (ONJ), atrial fibrillation, and atypical femur fracture [5, 6]. A novel therapy is possible with the advancement of stem cell-based therapy, which has been confirmed to reduce the susceptibility to fractures and improve the density of lost minerals by either increasing the number of resident stem cells or inducing them to proliferate and differentiate into bone-forming cells [7–12]. Nevertheless, living cells are unstable and thus the preservation of stem cells is an obstacle for further application [9, 13, 14]. In addition, living cells-based treatment normally does not offer off-the-shelf options. Thus, a novel therapeutic strategy is highly desirable to prevent and control osteoporosis.

**Scheme 1:**
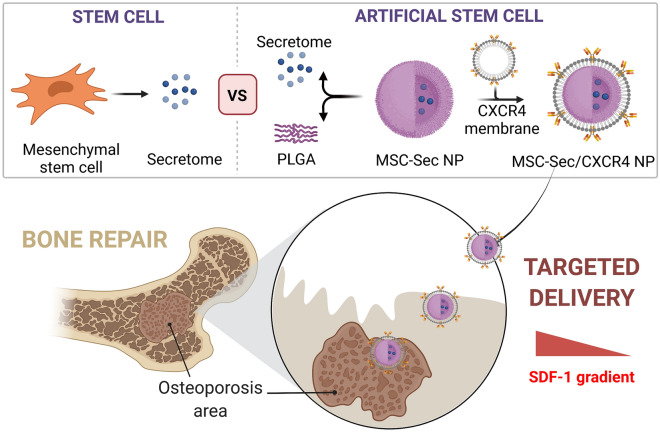
Composition/structure of stem cell mimicking MSC-Sec/CXCR4 NPs and schematic showing the concept of using MSC-Sec/CXCR4 NPs in the treatment of osteoporosis

Growing evidence indicates that most transplanted stem cells do not engraft into the recipient organs but instead exert their function by paracrine effects [15–20]. Stem cells release cytokines, chemokines, growth factors, and different types of extracellular vesicles (EVs) as part of their paracrine signaling system. To mimic the biofunction of stem cells, we and other groups have developed synthetic stem cells by loading conditioned medium to coat the cell membrane for injured heart or liver regeneration [21–25]. We have confirmed the synthetic stem cells’ superior stability even after repeated freeze–thaw cycles while maintaining high therapeutic efficiency. In this work, we synthesized mesenchymal stem cell (MSC) secretomes (Sec)-loaded polylactic-co-glycolic acid (PLGA) nanoparticles (MSC-Sec NPs) to mimic the real stem cells (Scheme 1). Furthermore, previous studies have indicated that MSCs possess the ability to sense the injured tissue and migrate to the damaged area via the interaction between chemo-attractant stromal cell-derived factor 1 (SDF-1) and its receptor chemokine receptor type 4 (CXCR4); blocking CXCR4 inhibited MSC migration. By taking advantage of the homing and engraftment capacity induced by the SDF-1/CXCR4 axis [26–28], we coated MSC-Sec NPs with human microvascular endothelial cell (HMEC) membranes, which have high levels of CXCR4 (MSC-Sec/CXCR4 NPs) for the treatment of osteoporosis. Furthermore, prostaglandin E2 (PGE2) has been reported to increase the expression level of CXCR4 on cell membranes [29], which could enhance their homing to bone marrow. We expected MSC-Sec/CXCR4 NPs to sense SDF-1 and migrate to the site of damage, where they release loaded paracrine molecules and can reverse osteoporosis.

## Results and discussion

### Fabrication and characterization of MSC-Sec/CXCR4 NPs

To mimic the biofunction of MSCs, we first synthesized MSC conditioned medium-encapsulated PLGA nanoparticles (MSC-Sec NPs). Many studies have confirmed that stem cell therapy is mainly attributed to paracrine effects. Stem cell-secreted factors such as osteoprotegerin (OPG, a potent inhibitor of osteoclastogenesis) and bone morphogenetic protein-2 (BMP-2, an inducer for osteoblast differentiation), promote healing and tissue repair [30–32]. Thus, the Sec of MSCs is expected to promote osteogenesis and inhibit osteoclasts, making Sec a potential treatment option for osteoporosis. First, we quantified the concentration of OPG and BMP-2 in Sec via ELISA, resulting in 17.5 pg per μg of protein and 1.7 pg per μg protein, respectively under normal oxygen culture (Additional file 1: Table S1). In addition, because the culture conditions may have an effect on the concentration of secreted factors, we quantified the concentration of OPG and BMP-2 under hypoxia, and the results, 18.1 pg OPG per μg protein and 2.7 pg BMP-2 per μg protein, showed that there was no significant difference between the concentrations under the two conditions. Then, we encapsulated Sec into PLGA nanoparticles with loading capacity and efficiency at 3.3% and 84.2% respectively, showing successful encapsulation of Sec into the hydrophilic core of the PLGA nanoparticles. After that, MSC-Sec NPs were further coated with HMEC membranes with overexpressed CXCR4 to form stem cell-mimicking particles with osteoporosis targeting ability (MSC-Sec/CXCR4 NP). First, we detected the expression level of CXCR4 on the surface of HMECs. Confocal imaging showed increased expression of CXCR4 after incubation with bFGF and prostaglandin E2 (PGE2). We also checked whether hypoxia conditions of the cell culture had any effect on the expression level of CXCR4 and found no significant change in the level of CXCR4. Flow cytometry results were consistent with the confocal imaging results (Additional file 1: Fig. S1).

**Fig. 1:**
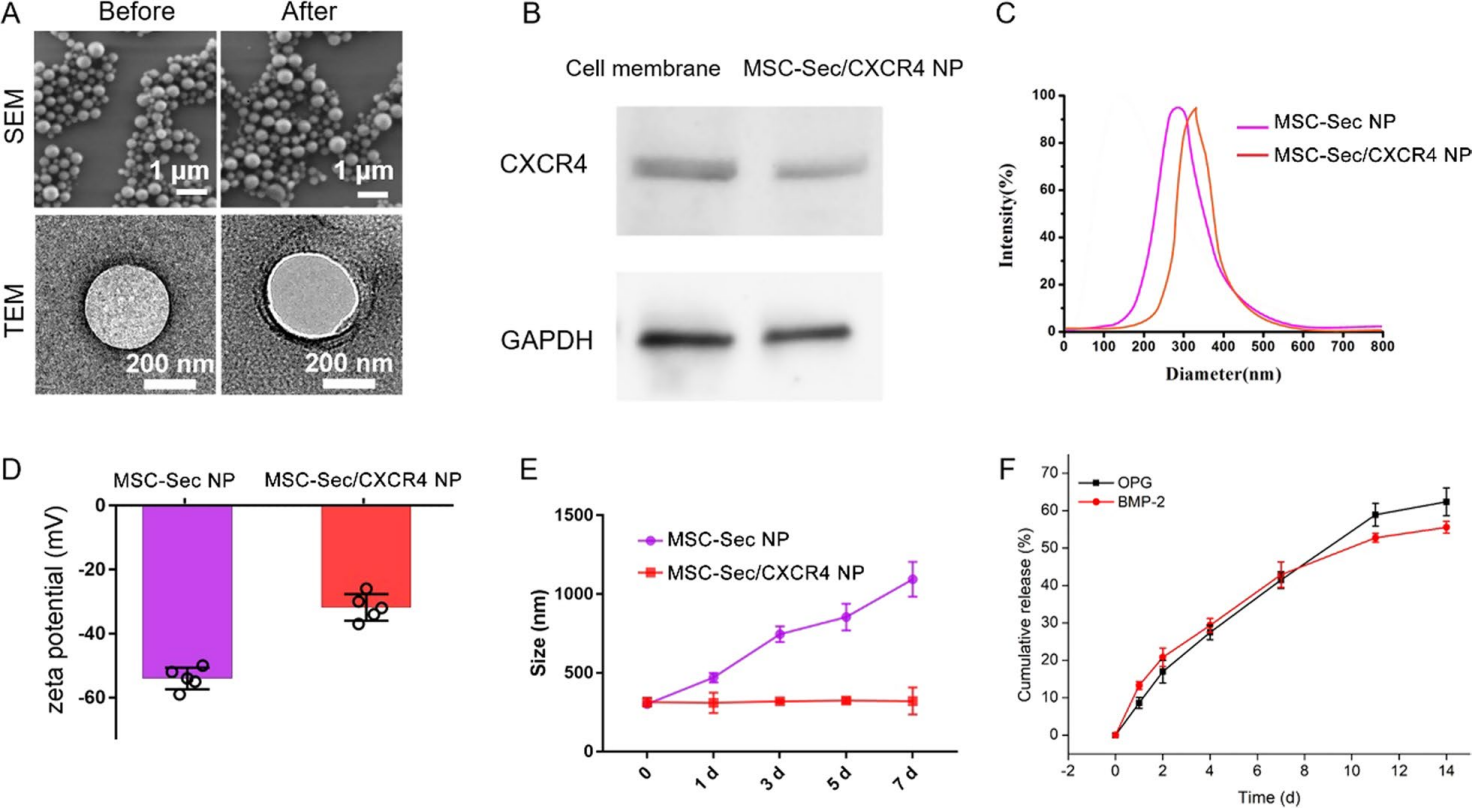
Fabrication and characterization of MSC-Sec/CXCR4 NP. **A** SEM and TEM images before and after cell membrane coating. **B** Western blot analysis detecting CXCR4 on MSC-Sec/CXCR4 NP. **C** Hydrodynamic diameter and **D** zeta potential of particles before and after membrane coating. **E** The stability of MSC-Sec NP and MSC-Sec/CXCR4 NP after storage in 50% fetal bovine serum at room temperature for days. **F** Quantitative analyses on the release of OPG and BMP-2 at different time points

The successful coating with HMEC membrane was first confirmed using scanning electron microscope (SEM) and transmission electron microscope (TEM) (Fig. 1A). According to SEM results, the PLGA nanoparticles were around 300 nm before and after coating. However, we cannot identify the membrane after coating using SEM. We were able to see a hazy layer around the particles from TEM images, indicating the presence of membrane on the surface. In addition, western blots were performed to confirm the CXCR4 membrane coating (Fig. 1B). As with membrane alone, the membrane-coated particles were found to have a CXCR4 band. We also studied the size and zeta potential change before and after coating. As shown in Fig. 1C, the size of the nanoparticles showed a slight increase after HMEC membrane coating, while the zeta potential of MSC-Sec/CXCR4 NP changed from − 52 to − 29 mV (Fig. 1D). The stability of MSC-Sec/CXCR4 NP in 10% serum showed no obvious change for the duration of one week-incubation, indicating the stability of MSC-Sec/CXCR4 NP and their potential for further application (Fig. 1E). In contrast, MSC-Sec NP without membrane coating increased in size owing to agglomeration. Finally, the Sec release behavior of MSC-Sec/CXCR4 NP was investigated and we found they could sustain the release of growth factors OPG and BMP-2 for about two weeks (Fig. 1F). This sustained release is promising for osteoporosis treatment.

### MSC-Sec/CXCR4 NPs inhibit receptor activator of nuclear factor kappa-B Ligand (RANKL)

Osteoblastic RANKL promotes differentiation by triggering RANKL reverse signaling, which activates runt-related transcription factor 2 [33]. Cultures supplemented with recombinant RANKL have been widely used to promote macrophage osteoclast differentiation. We first isolated rat bone marrow macrophages (rBMMs) and induced osteoclast formation by supplementing with recombinant RANKL with or without our MSC-Sec/CXCR4 NPs. We found that introduction of 10^4^ MSC-Sec/CXCR4 NP resulted in macrophage differentiation into atypical osteoclasts, and 10^6^ MSC-Sec/CXCR4 NP successfully inhibited RANKL-induced macrophage osteoclast differentiation altogether, indicating that MSC-Sec/CXCR4 NP have an inhibiting effect on osteoclast formation (Additional file 1: Fig. S2).

### MSC-Sec/CXCR4 NPs promote the proliferation of MSCs and osteoblasts

**Fig. 2:**
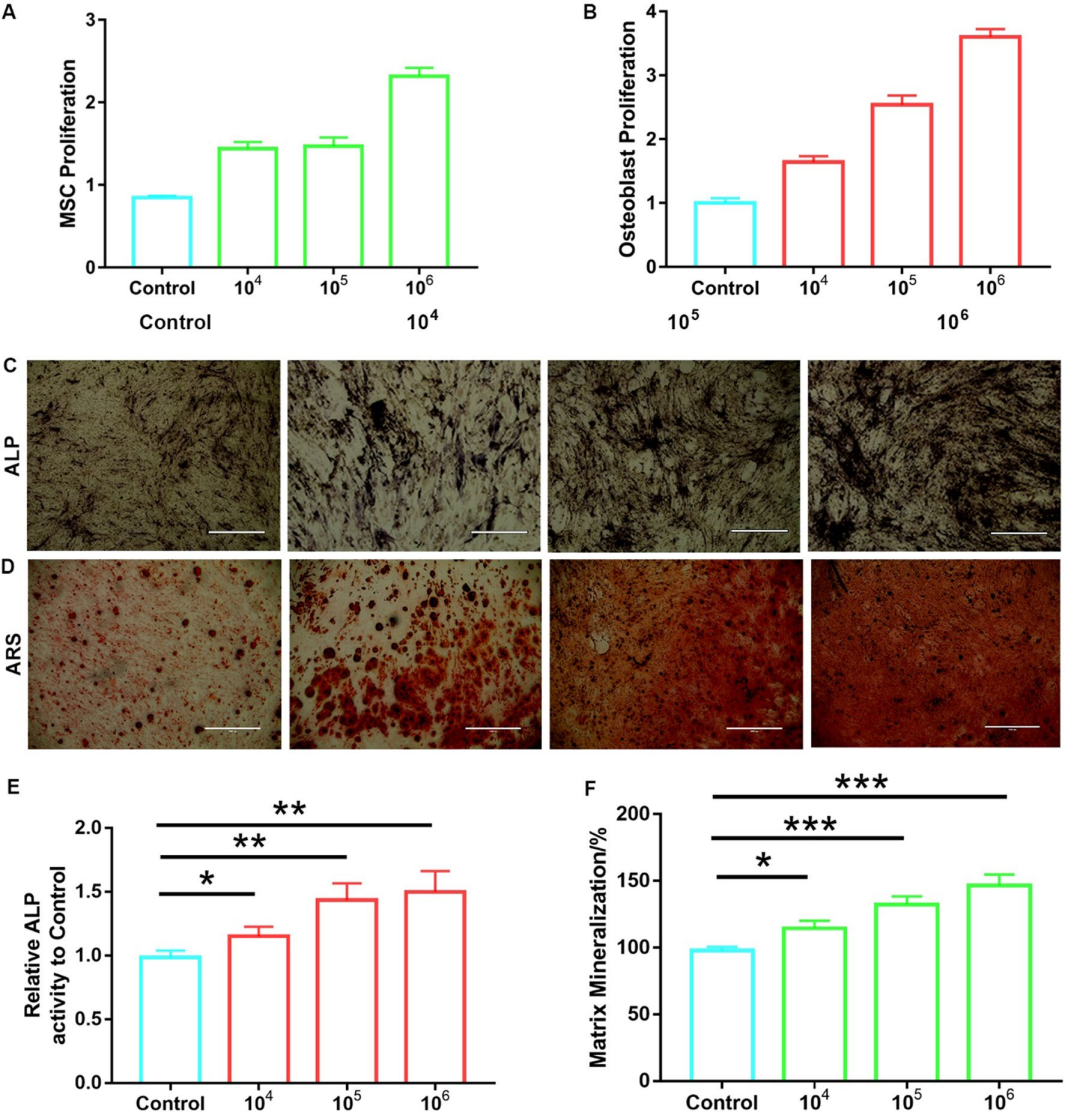
MSC-Sec/CXCR4 NP inhibits osteoclast differentiation and promotes osteogenic proliferation. Cell proliferation for **A** MSCs and **B** osteoblasts that were incubated with different numbers of MSC-Sec/CXCR4 NPs. **C** ALP activity and **D** Alizarin Red S staining of primary MSCs after incubating with different concentrations of MSC-Sec/CXCR4 NPs for 2 weeks. Scale bar, 1 mm. Quantifying **E** relative ALP activity and **F** the amount of Alizarin Red S that stained the mineralized matrix. *p < 0.05, ** p < 0.01 and *** p < 0.001

Bone formation needs to recruit a sufficient number and activity of osteoblasts. In addition, MSCs aid in fracture healing by properly proliferating, differentiating, and, consequently, forming bone. Thus, the proliferation of the two kinds of cells plays an important role for the treatment of osteoporosis. To test the ability of MSC-Sec/CXCR4 NPs to promote proliferation in vitro, MSCs and osteoblasts were co-cultured with MSC-Sec/CXCR4 NPs in different concentrations. As shown in Fig. 2A, B, MSC-Sec/CXCR4 NPs significantly promote the proliferation of MSCs and osteoblasts with concentration-dependent effects owing to the sustained release of Sec. As alkaline phosphatase (ALP) is an osteogenesis marker, we then measured ALP activity and found enhanced osteogenesis differentiation with enhanced concentrations of MSC-Sec/CXCR4 NPs (Fig. 2C, E). The formation of mineralized matrix nodules is a marker for late stages of osteogenesis; we also measured the matrix nodules of the MSCs after incubating with MSC-Sec/CXCR4 NPs for 2 weeks using the Alizarin Red S (ARS) staining assay. The results were consistent with Fig. 2C in that more matrix nodules were formed when treated by higher concentrations of particles (Fig. 2D, F).

### CXCR4 mediated MSC-Sec NPs targeting to bone of ovariectomized (OVX) rats

**Fig. 3:**
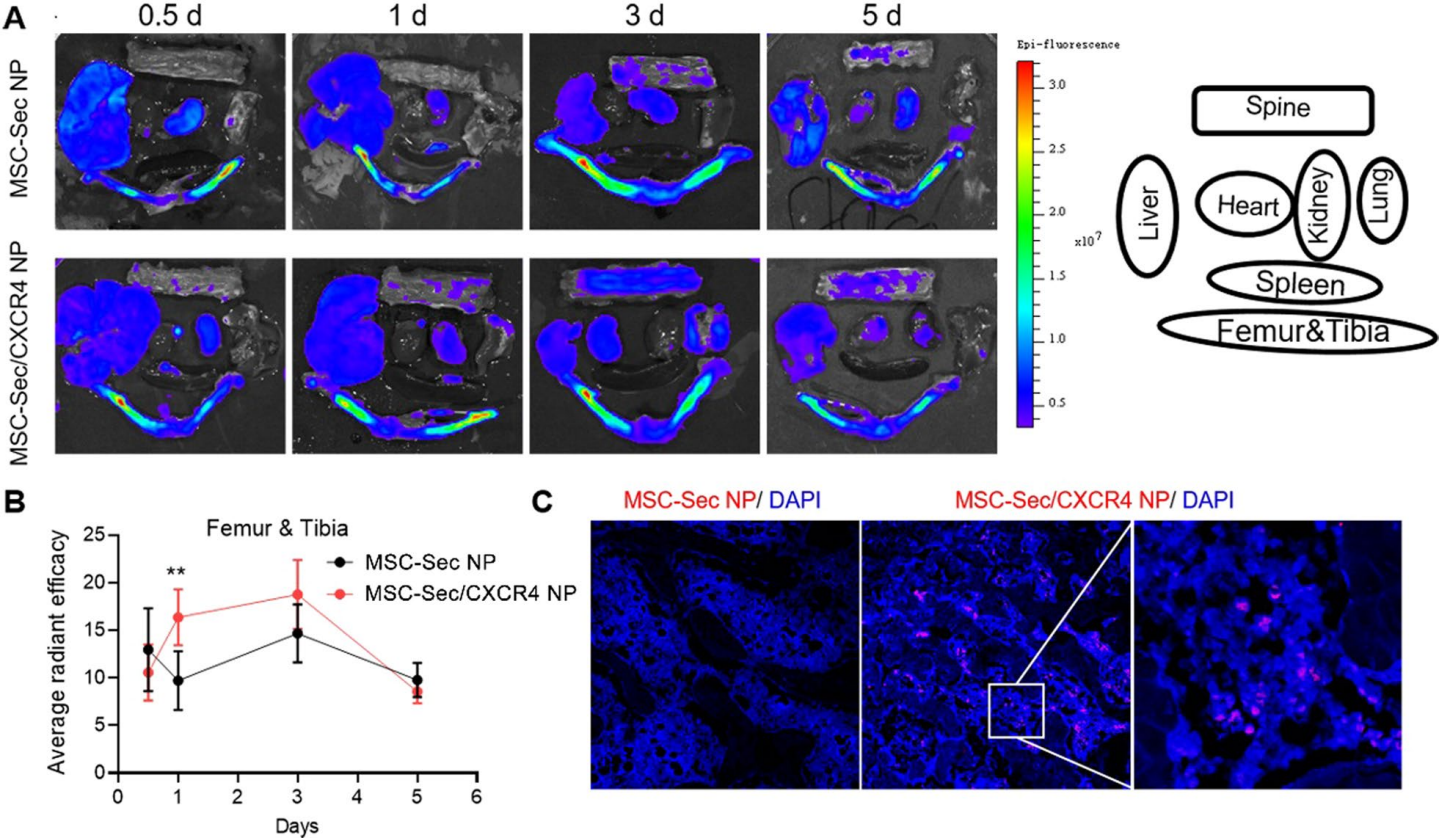
MSC-Sec/CXCR4 NPs targets the bone. **A** Biodistributions of rhodamine B-labelled MSC-Sec NPs with or without CXCR4 membrane coating after systemic administration in OVX rat at different time points. **B** Quantitative analysis of fluorescent intensities of femur and tibia at different time points post intravenous rhodamine B-labelled MSC-Sec NPs with or without CXCR4 membrane coating. **C** Confocal imaging of MSC-Sec/CXCR4 NPs in massive bone at day 1 after infusion

Previous studies have indicated that both CXCR4 positive cells and CXCR4-incorporated particles have homing-related responses to the SDF-1 gradient, which is highly secreted during ischemia, inflammation, or osteoporosis. To test the osteoporosis-targeting ability of MSC-Sec/CXCR4 NPs in vivo, an osteoporosis rat model was constructed by ovariectomy (OVX). We monitored the biodistribution of rhodamine B (RhB)-labelled MSC-Sec/CXCR4 NPs and bare MSC-Sec NPs at different time points in OVX rats. As shown in Fig. 3A and B, more RhB@MSC-Sec/CXCR4 NPs accumulated in the femur on day 3 and remained as long as day 5, indicating the bone-targeting ability of CXCR4 membrane-incorporated particles. In contrast, MSC-Sec NPs alone resulted in a slight accumulation in the femur. In addition, more RhB@MSC-Sec/CXCR4 NPs were detected in bone than bare MSC-Sec NPs, indicating the superior ability of MSC-Sec/CXCR4 NPs respond to the SDF-1 gradient (Fig. 3C).

### MSC-Sec/CXCR4 NPs relieve bone mass attenuation in OVX rats

**Fig. 4:**
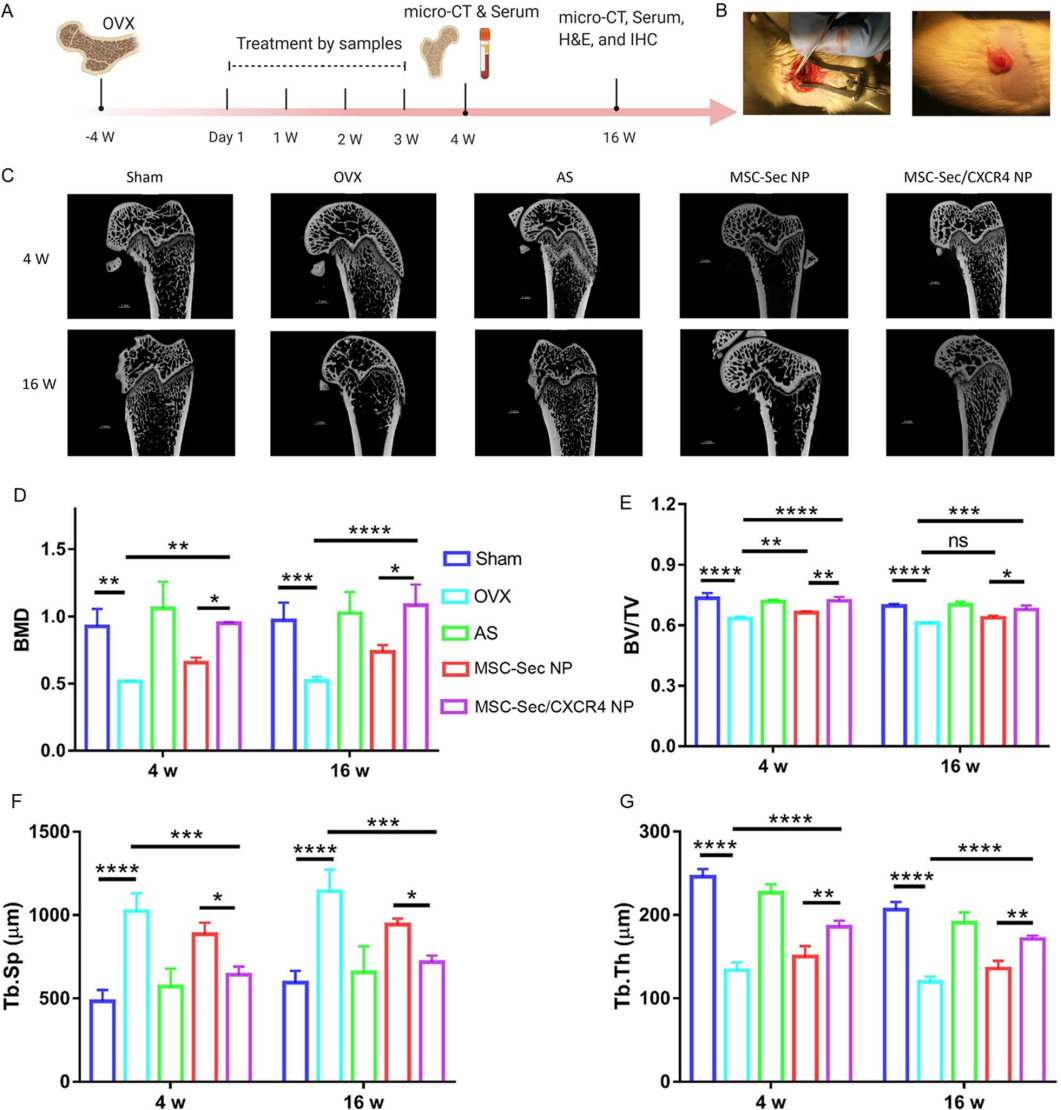
MSC-Sec/CXCR4 NPs reduces OVX-induced osteoporosis in rats. **A** Schematic showing the design of in vivo experiments in rats with osteoporosis. **B** Photograph of OVX surgery to induce osteoporosis. **C** micro-CT images of distal femur after treating with different samples at 4 week and 16 weeks. Quantitative results of **D** BMD changes and some trabecular parameters, including trabecular bone volume expressed as percentage of **E** total tissue volume (BV/TV), **F** trabecular thickness (Tb.Th), and **G** trabecular separation (Tb.Sp). n = 6 rats per group. *p < 0.05, **p < 0.01, ***p < 0.001, and ****p < 0.0001

To study the therapeutic effects of injected MSC-Sec/CXCR4 NPs, we performed ex vivo micro-computed tomography (Micro-CT) to detect proximal tibiae at 4- and 16-weeks post-treatment (Fig. 4A). The osteoporosis rat model induced by OVX was used for the animal study (Fig. 4B). CT images accurately showed bone microstructural parameters for quantitative assessments (Fig. 4C). As shown in Fig. 4D-G, we found increased bone mineral density (BMD), trabecular bone volume, and trabecular thickness in the MSC-Sec/CXCR4 NPs group compared to the OVX group (control group). In contrast, we found trabecular separation was reduced. Interestingly, compared with the MSC-Sec NPs treatment group, MSC-Sec/CXCR4 NPs induced higher trabecular bone volume, thickness, and number, which was comparable with the alendronate sodium (AS)-treated group owing to CXCR4 induced targeting effect. Consistent with Micro-CT results, we detected a significant increase in BMD in rats treated with MSC-Sec/CXCR4 NPs when compared to the OVX group or the MSC-Sec NPs group.

### MSC-Sec/CXCR4 NPs promote bone formation and inhibit bone resorption

**Fig. 5:**
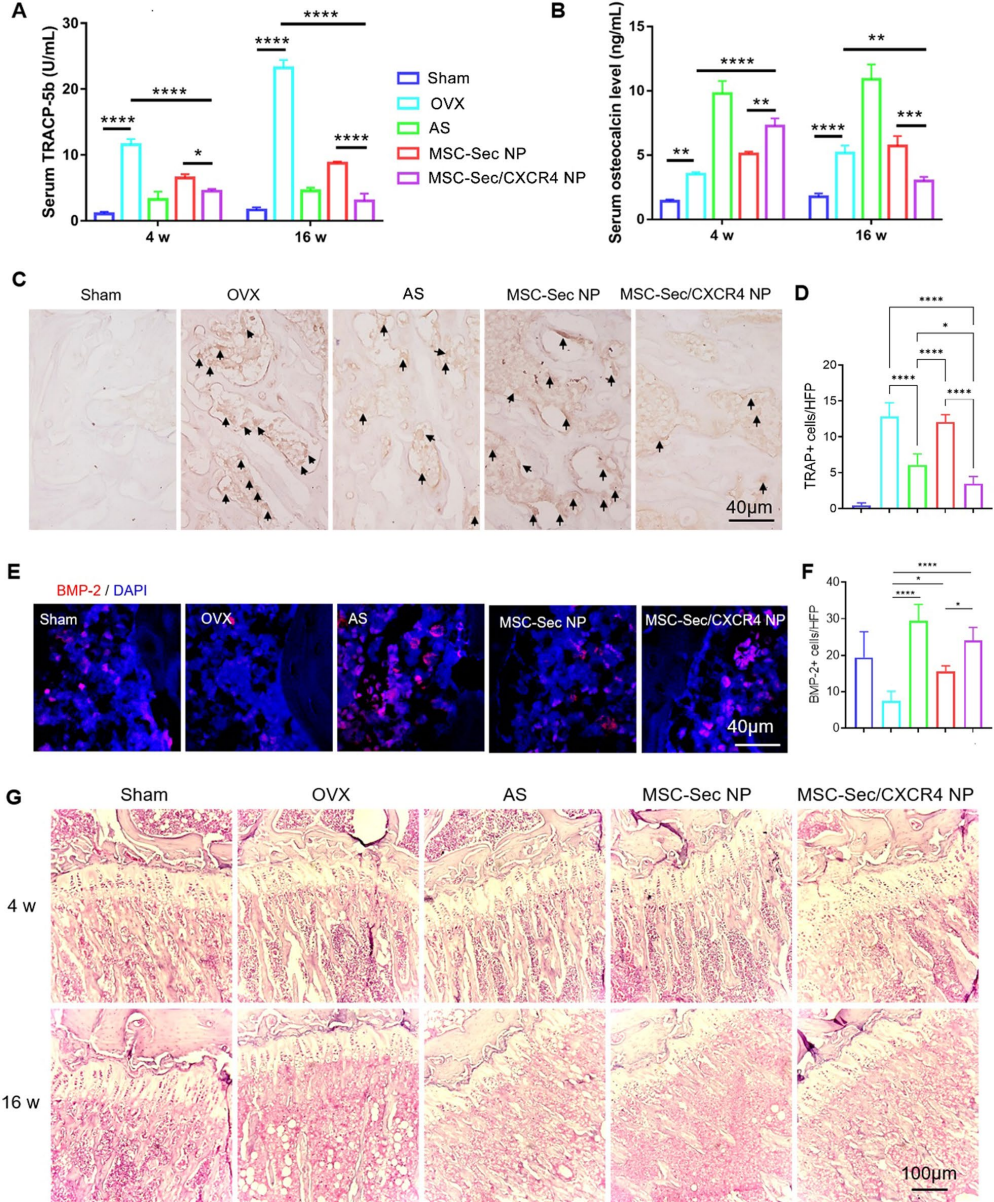
MSC-Sec/CXCR4 NPs therapy promotes bone formation and inhibits bone resorption. **A** Detection of serum TRACP-5b and **B** osteocalcin level after 4-week and 16-week treatment. **C** TRAP staining showing the osteoclasts. **D** Quantification of osteoclasts cells on the bone surface. **E** Detection of BMP-2 after 4-week and 16-week treatment and **F** their quantitative results. **G** Photomicrographs of bone sections stained by H&E. n = 6 rats per group. *p < 0.05, **p < 0.01, ***p < 0.001, and ****p < 0.0001

Percent changes in serum tartrate-resistant acid phosphatase (TRACP)-5b (a marker of bone resorption) and osteocalcin (a specific biochemical parameter of bone formation) could estimate the therapeutic effect. Thus, we examined the concentration of TRACP-5b and osteocalcin in serum after different treatments using an enzyme-linked immunosorbent assay. MSC-Sec/CXCR4 NPs, AS, and MSC-Sec NP treatment markedly decreased serum levels of TRACP-5b at 4 weeks post treatment and up to 16 weeks post treatment (Fig. 5A). In addition, TRACP-5b level was lower in the MSC-Sec/CXCR4 NPs group compared to the MSC-Sec NPs group and showed comparable therapeutic efficiency to the AS treatment group. Furthermore, our tartrate-resistant acid phosphatase (TRAP) staining results were consistent with serum results that MSC-Sec/CXCR4 NPs reduced the TRAP level, indicating reduced osteoclast numbers (Fig. 5C, D). We also tested the osteocalcin to see the enhanced bone formation effect induced by MSC-Sec/CXCR4 NPs (Fig. 5B). Compared with the sham group, OVX passively promoted osteogenesis, which resulted in the slight increase of osteocalcin. In contrast, after 4 weeks post-treatment, osteocalcin level was significantly increased compared to pretreatment, and the MSC-Sec/CXCR4 NPs group showed the most efficiency. BMP-2 contributes to bone formation, and we found increased BMP-2 expression in MSC-Sec/CXCR4 NPs treated rats after targeted delivery of conditioned medium (Fig. 5E, F). In addition, H&E staining showed massive bone loss after OVX surgery. Injection of MSC-Sec NPs increased bone volume while treatment with CXCR4-MSC-Sec NPs further improved the outcomes (Fig. 5G). Taken together, these results indicate MSC-Sec/CXCR4 NPs could promote bone formation while inhibiting bone resorption, which then reverses osteoporosis.

## Conclusion

Osteoporosis is a chronic condition that generally requires sustained medical interventions to limit the risk of additional bone loss. Stem cells have been studied for osteoporosis treatment. Unlike traditional chemical drugs, stem cells are live drugs that can proliferate and differentiate in the living body, and thus only one dose is needed [34]. However, osteoporosis is a systemic disease and systemic intravenous injection poses the risk of inducing blood clots. In addition, stem cells are usually unstable, and hard to preserve and transport. Regardless, prior to clinical application, systemic studies on the effect of dose, bioavailability, and pharmacokinetics of stem cells need to be performed. Biomaterials or biomimetic materials have been widely developed for regenerative medicine owing to their stability, biocompatibility, and multifunctionality [35–37]. Synergizing the benefits of both stem cells and biomaterials, in this work, we fabricated a synthetic stem cell nanoparticle for osteoporosis treatment using CXCR4-expressing, membrane-coated, and secretome-loaded PLGA nanoparticles. The CXCR4/SDF-1 axis has been established to modulate stem cell migration to injured tissue. CXCR4-overexpressing membrane-coated particles for targeted delivery have been developed. For examples, Ma et al. reported neural stem cell membrane-coated PLGA for ischemic brain-targeted delivery and Luo et al. used engineered primary mouse thoracic aorta endothelial cell membrane and rapamycin-loaded nanoparticles for targeting and repairing cerebral ischemia–reperfusion (I/R) injury [38, 39].

We have confirmed that the stem cell secretome includes OPG and BMP-2, two important factors in osteoporosis therapy. Just like real stem cells, our MSC-Sec/CXCR4 NP showed sustained and long-term (at least 14 days) release of OPG and BMP-2 (Fig. 1F) and 10^6^ of MSC-Sec/CXCR4 NP inhibited osteoclast differentiation (Additional file 1: Fig. S2) while promoting osteogenesis (Fig. 2). The presence of CXCR4 on MSC-Sec/CXCR4 NP ensures the synthetic nanoparticles’ mimicking of stem cell accumulation in the bone microenvironment due to their interaction with SDF-1 (Fig. 3).

Using micro-CT analysis, we found that injection of MSC-Sec/CXCR4 NP reduces OVX-induced bone mass attenuation in OVX rats. CXCR4 enhanced the accumulation of MSC-Sec/CXCR4 NP and thus resulted in a higher concentration of OPG and BMP-2 in the bone microenvironment, which then efficiently promoted osteogenesis and inhibition of osteoclasts. As expected, fewer osteoclasts were found after MSC-Sec/CXCR4 NP treatment (Fig. 5C) and there was an increased bone volume indicated by H&E staining (Fig. 5G). In addition, the level of TRACP-5b was lower and osteocalcin secreted solely by osteoblasts was higher in serum treated with MSC-Sec/CXCR4 NP (Fig. 5A, B). Although MSC-Sec/CXCR4 NP achieved a final therapeutic effect comparable to alendronate sodium, they can potentially avoid the side effects associated with alendronate sodium.

In conclusion, we have confirmed that synthetic stem cells have potential as a therapeutic drug in the treatment of chronic osteoporosis with sustained and long-term drug releasing behavior.

## Supplementary Information

**Additional file 1.** **Table S1.** Quantitative results of OPG and BMP-2 in MSC conditioned medium. **Fig. S1.** Representative confocal fluorescent images showing CXCR4-positive HMEC and flow cytometry analysis. **Fig. S2.** Images taken from isolated rat bone marrow macrophages (rBMMs) after induced differentiation into osteoclasts by RANKL in the presence of PBS, 10^4^ MSC-Sec/CXCR4 NP or 10^6^ MSC-Sec/CXCR4 NP.

## Data Availability

All data generated or analyzed during this study are included in the article.
