# Supplementary material for: Nanoparticles functionalized with stem cell secretome and CXCR4-overexpressing endothelial membrane for targeted osteoporosis therapy
Source: J Nanobiotechnology. 2022 Jan 15;20:35. doi: 10.1186/s12951-021-01231-6 (PMC8760699; doi:10.1186/s12951-021-01231-6)
Supplement: Supplementary file 1 — Additional file 1. Table S1. Quantitative results of OPG and BMP-2 in MSC conditioned medium. Fig. S1. Representative confocal fluorescent images showing CXCR4-positive HMEC and flow cytometry analysis. Fig. S2. Images taken from isolated rat bone marrow macrophages (rBMMs) after induced differentiation into osteoclasts by RANKL in the presence of PBS, 104 MSC-Sec/CXCR4 NP or 106 MSC-Sec/CXCR4 NP. [file 12951_2021_1231_MOESM1_ESM.docx]

**Additional file**

**Nanoparticles functionalized with stem cell secretome and CXCR4-overexpressing endothelial membrane for targeted osteoporosis therapy**

Chi Zhang^1,2#^, Wei Zhang^1#^, Dashuai Zhu^2#^, Zhenhua Li^2^, Zhenzhen Wang^2^, Junlang Li^2^, Xuan Mei^2^, Wei Xu^3*^, Ke Cheng^2*^, Biao Zhong^1*^

^1^Department of Orthopedics, Shanghai Jiao Tong University Affiliated Sixth People's Hospital, 600 Yishan Road, Shanghai 200233, China.

^2^Joint Department of Biomedical Engineering, the University of North Carolina at Chapel Hill and North Carolina State University, North Carolina, USA.

^3^Department of Orthopedics, Tongren Hospital, Shanghai Jiao Tong University School of Medicine, 1111 XianXia Road, Shanghai 200336, China

*Corresponding author. E-mail: biao.zhong@sjtu.edu.cn, kcheng3@ncsu.edu, weixu@shsmu.edu.cn

^#^Chi Zhang, Wei Zhang, and Dashuai Zhu contributed equally to this work.

**Materials and methods**

**Preparation of Bone Marrow-derived Mesenchymal Stem Cells (BMSCs) Secretomes-Loaded PLGA nanoparticles (MSC-Sec NP)**

Human BMSCs were directly obtained from ATCC (cat no.: 63208778). The cells were cultured per vendor’s instructions. Briefly, the MSCs were cultured in Iscove’s modified Dulbecco’s medium (IMDM, Thermo Fisher Scientific) for 3 days, and then the supernatant was collected to harvest secretomes. Conditioned medium was collected and filtered through a 0.22 μM filter into a sterile 50 mL conical to remove any cell debris and contaminants. Sterile conditioned media was stored at −80°C for at least 24 h then lyophilized by a freeze-dry system. MSC-Sec NP were fabricated by a double emulsion process followed by membrane extrusion. In brief, the first emulsion (w_1_/o) was prepared by dispersing 0.16 mL of secretome solution (1 %, w/v) in 1.51 mL of DCM containing PLGA (250 mg) and Span 60 (3.5, 7, 10, and 14 %, w/w) solution. The mixture was sonicated for 15 s for emulsification at 40 W output power in an ice cooling bath to prevent the temperature from rising. The resulting emulsion was added gradually to 5 mL of an aqueous solution containing PVA (1 %, w/v) with Tween 80 as a surfactant in different percentages (0, 4, 8, and 16 %, w/v), then sonicated for 15 s under the same conditions to form the second emulsion (w_1_/o/w_2_). Constant stirring of the resulting emulsion was maintained at 1200 rpm under ambient temperature for about 3 h until the DCM had completed evaporation. The prepared nanoparticles were harvested and washed by five successive ultracentrifugations in deionized water at 9,000 rpm for 20 min. The particles were then extruded through membranes with pore sizes of 400 nm using an extruder (Avanti Polar Lipids, Alabaster, AL). The final product was stored in a desiccator at −20 °C until use. Blank nanoparticles without secretomes were prepared in the same manner and used as control samples throughout this research.

**Generation of CXCR4-included human microvascular endothelial cells (HMECs) Membrane Vesicles**

HMECs were purchased from Cell Biologics, Inc. (Catalog No. H-6220). Cells were cultured in T25 tissue culture flasks pre-coated with gelatin-based solution for 2 min, and incubated in Cell Biologics’ Culture Complete Growth Medium (Catalog No. H1168) generally for 3-7 days. To obtain CXCR4-overexpressed cell membrane, HMECs were stimulated with VEGF (10 ng/mL), bFGF (50 ng/mL), and different concentrations of PGE2 ranging from 10^-6^ to 10^-11^ M. Optimal doses for PGE2 were then used in a kinetic assay at 24, 48, and 72 hours for cell surface expression of CXCR4 by immunofluorescence. To characterize the CXCR4 on HMECs in hypoxia and normoxia, flow cytometry (Beckman Coulter flow cytometer) and confocal microscopy were performed.

To prepare HMEC membrane vesicles, HMEC shells were subjected to three freeze/thaw cycles. After that, the collected HMEC shells were extruded through membranes with pore sizes of 400 and 200 nm using an extruder (Avanti Polar Lipids).

**Fabrication and Characterization of CXCR4 Membrane-coated MSC-Sec NP (MSC-Sec/CXCR4 NP)**

To cloak the CXCR4 overexpressed cell membrane vesicles onto the surface of MSC-Sec NP, 0.5 mL of MSC-Sec NP (1×10^9^/mL) was mixed with 0.5 mL CXCR4 membrane vesicles (1×10^9^/mL) and then extruded 11 times. The resulting MSC-Sec/CXCR4 NP were centrifuged at 800 g to remove excess membrane debris. Nanoparticle concentration and size were examined by NanoSight (Malvern, UK). Surface charge (ζ potential, mV) was measured by dynamic light scattering (DLS). The morphology of MSC-Sec NP-CXCR4 was studied by TEM (JEOL JEM-2000FX). The coated specimen was imaged after negative staining with 1 wt% uranyl acetate. To reveal whether MSC-Sec/CXCR4 NP have CXCR4 as HMEC membrane, western blot was performed to reveal the CXCR4 on both membrane and the dialyzed MSC-Sec/CXCR4 NPs. To evaluate the stability in serum, the different nanoformulations were incubated with 50% fetal bovine serum (Hyclone, USA). Long-term stability was assessed by the particle size change measured by DLS before lyophilization in 10 wt% sucrose and after resuspension in PBS back to the original volume.

**Growth Factor Release Study**

Total protein and growth factor release from MSC-Sec/CXCR4 NP were determined using ELISA. In brief, freeze-dried MSC-Sec/CXCR4 NP were dissolved in DCM. After that, PBS was added to the solution. The sample was vortexed for 5 min to isolate proteins from the oil phase to the water phase. After centrifugation, the protein concentration in the water phase was measured by BCA protein assay. For growth factor release studies, nanoparticles were incubated in PBS at 37 °C. The supernatant was collected at various time points (day 1, 3, 5, 7, 11, and 14) after centrifugation at 20000 g for 30 min to pellet the nanoparticles. The concentrations of osteoprotegerin (OPG) and BMP-2 were measured using ELISA kits according to the manufacturer’s instructions. The data were averaged from three independent measurements.

**Study of Proliferation of MSCs and Osteoblasts induced by MSC-Sec/CXCR4 NPs**

MSCs or osteoblasts were cultured on 96-well plates for 3 d, followed by co-incubation with 10^4^, 10^5^, or 10^6^ MSC-Sec/CXCR4 NP for 48 h. After that, cell proliferation was detected using the Cell Counting Kit-8 (CCK-8) assay.

**ALP activity**

MSCs (2.5 × 10^6^ cells per well) were seeded and cultured in 48-well culture plates. The cells were treated with different concentrations of MSC-Sec/CXCR4 NPs. Then, 5.0 mM β- glycerophosphate and 50 μg /mL ascorbic acid were added to the medium. After being exposed to samples for 2 weeks, the cells were rinsed with deionized water, fixed with 4% paraformaldehyde, and stained with an alkaline phosphatase (ALP) kit (ab83369) for further imaging. In addition, cells were also washed with ice-cold PBS and lysed. ALP activity was evaluated by an ALP activity kit according to the manufacturer's instructions.

**ARS staining**

The formation of mineralized matrix nodules was measured as a marker at the later time points of osteogenesis. The mineralized extracellular matrix was stained with ARS to determine bone mineralization. MSCs (5×10^6^ cells per well) were seeded in a 24-well culture plates. After being exposed to samples for 2 weeks, the cells were fixed with 95% ethanol for 10 min at 37°C, washed with PBS, stained with 40 mM ARS for 30 min, and washed with deionized water. Quantitation of mineralized matrix nodules was carried out by dissolving matrix nodules with 10% (w/v) cetylpyridium chloride and measuring the absorbance values at 570 nm.

**The Ovariectomized (OVX) Rat Model.**

All animal work was compliant with the Institutional Animal Care and Use Committee (IACUC) of the University of North Carolina at Chapel Hill and North Carolina State University. The ovaries from six- to eight-month-old female SD rats (n = 6) were removed and the rats were left for four weeks after surgery before measuring their bone mineral density (BMD) using microCT (Bruker micro-CT system, Germany). The rats were intravenously injected with 0.5 mL PBS (PBS control group) or 10^7^ MSC-Sec NPs or MSC-Sec/CXCR4 NPs in 0.5 mL PBS at day 1, day 8, day 15, and day 22 following OVX. In addition, alendronate sodium (0.25 mg/kg) was used as a control.

**Evaluation of the In Vivo Targeting Ability of MSC-Sec/CXCR4 NP**

Rhodamine B (RhB)-labeled PLGA was used to synthesize MSC-Sec NPs and MSC-Sec/CXCR4 NPs. RhB-labeled MSC-Sec NP and MSC-Sec/CXCR4 NP were iv administrated to OVX rats. The rats were euthanatized at different time points (0.5 d, 1 d, 3 d, and 5 d) and the major organs (heart, liver, spleen, lung, kidney, femur, and tibia) were harvested for in vivo imaging.

**Blood Collection and Serum Analysis**

OVX rats were killed at 4 weeks or 16 weeks, and the blood was collected from the venous cava immediately. For serum collection, harvested blood was allowed to clot at room temperature for 30 minutes. Serum was aspirated from the supernatant after centrifugation at 1000 g, 4°C for 15 minutes. The levels of TRACP-5b (E-EL-R0939, Elabscience) and osteocalcin (NBP2-68153, Novus) were detected by commercially available ELISA kits.

**Immunostaining and Haematoxylin and Eosin Staining**

We fixed the right femurs in 4% buffered formalin for 24 hours and placed them in 9% formic acid for decalcification for 21 days. The sample was cut in the middle at a mid-sagittal plane and embedded in OCT. Samples were cut at 5 μm thickness. Slides were placed in methanol and TRAP staining was performed using a commercial acid phosphatase leukocyte kit (Sigma, St Louis, MO). Slides were also stained with Haematoxylin and Eosin (H&E) and immunohistochemical stain of BMP-2.

**Bone Morphometry and BMD Measurement**

To track new bone formation, calvarial bones of anesthetized rats were scanned and analyzed using microCT (Bruker micro-CT system, Germany) at 4- and 16-weeks post-surgery.

**Statistical Analysis**

All experiments were performed independently at least three times, and the results were presented as mean ± s.d. Comparisons between any two groups were performed using two-tailed, unpaired Student’s t-test. Comparisons between more than two groups were performed using one-way ANOVA, followed by post hoc Bonferroni test. Single, double, and triple asterisks represent p < 0.05, 0.01, and 0.001, respectively; p < 0.05 was considered statistically significant.

**Supplementary Tables and Figures**

**Table S1.** Quantitative results of OPG and BMP-2 in MSC conditioned medium.

|  | Hypoxia | Normoxia |
| --- | --- | --- |
| OPG | 18.1 pg per 1 μg protein | 17.5 pg per 1 μg protein |
| BMP-2 | 2.7 pg per 1 μg protein | 1.7 pg per 1 μg protein |


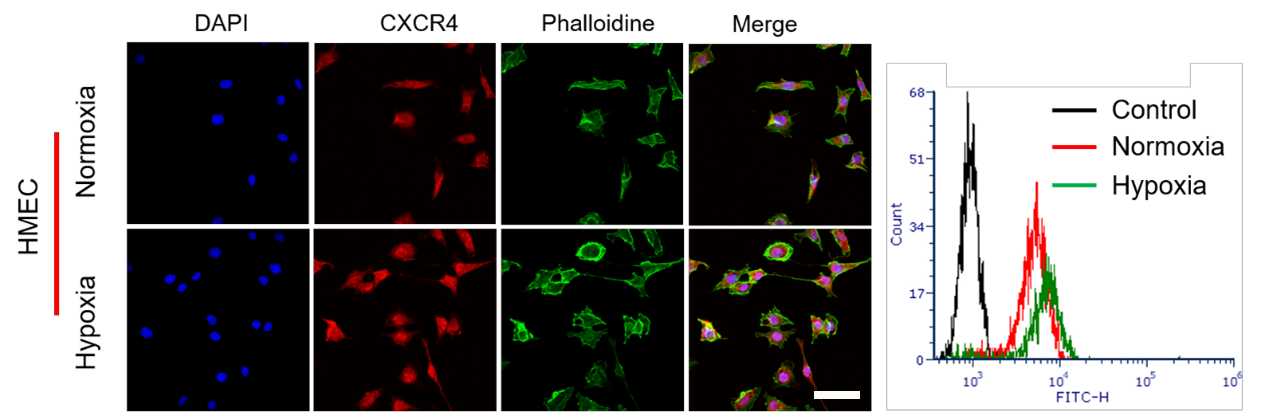


**Fig. S1** Representative confocal fluorescent images showing CXCR4-positive HMEC and flow cytometry analysis. Scale bar, 50 μm.


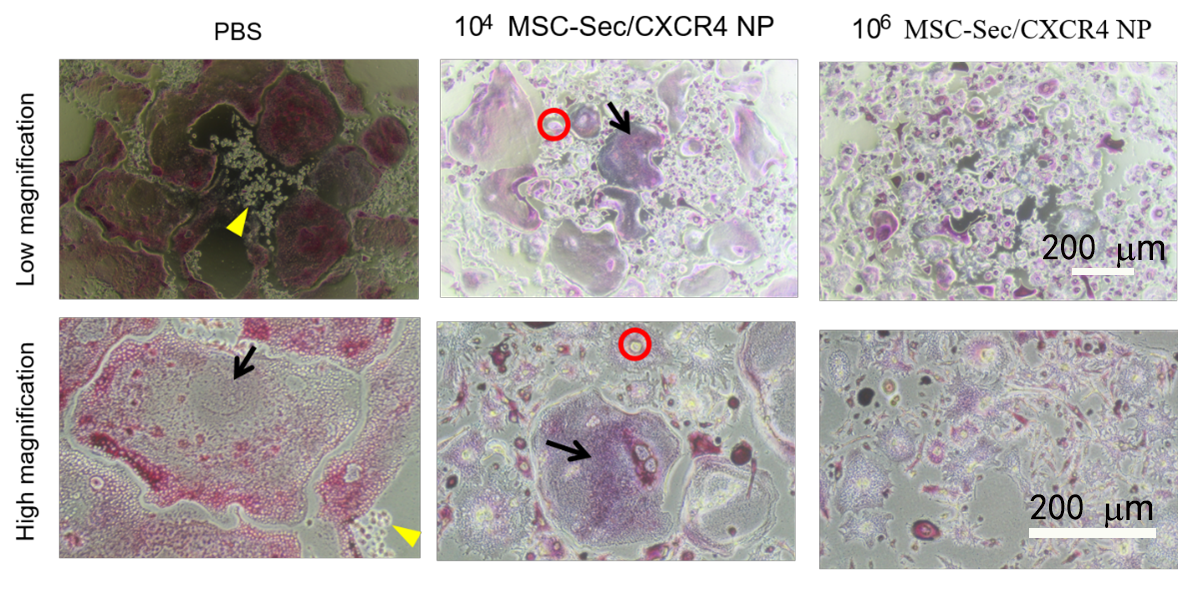


**Fig. S2** Images taken from isolated rat bone marrow macrophages (rBMMs) after induced differentiation into osteoclasts by RANKL in the presence of PBS, 10^4^ MSC-Sec/CXCR4 NP or 10^6^ MSC-Sec/CXCR4 NP. The black arrows indicate osteoclasts, the yellow triangles indicate macrophages, and the red circles indicate atypical osteoclasts.
